# Three Seizures Provoked by E-cigarette Use in a Five-Year Period: A Case Report

**DOI:** 10.7759/cureus.27616

**Published:** 2022-08-02

**Authors:** Jasmine A Liu-Zarzuela, Ruiqing Sun

**Affiliations:** 1 Neurology, University of Texas Medical Branch, Galveston, USA

**Keywords:** vaping, electronic cigarettes, nicotine, seizure, seizure risk

## Abstract

We report a case of three seizures provoked by e-cigarette use (vaping) within the time span of five years from youth to young adult. At presentation, the neurological exam was unremarkable. Computerized tomography (CT) of the head, magnetic resonance image (MRI) of the brain, electroencephalograms (EEG), electrocardiogram (EKG), and transthoracic echocardiogram (TTE) were normal. Multiple toxicology screens were normal as well. Each seizure occurred within minutes of vaping, thereby suggesting a temporal association and a possible causal relationship between e-cigarettes and seizures.

## Introduction

On June 24, 2022, The Food and Drug Administration (FDA) issued marketing denial orders to JUUL Lab Inc., a company that distributes and sells nicotine-containing devices and products, such as e-cigarettes and vape pens, in the United States. This order prohibits commercial distribution and sales of nicotine-containing products, but individuals can still own and use them [1,2]. While e-cigarette usage was initially advertised as a safe mechanism for smoking cessation, time has revealed its multitude of negative impacts on the human body, most notably on the respiratory, cardiovascular, and immune systems [2-6]. Research has recently focused on the effect of e-cigarette usage on the human brain. For example, nicotine can harm the developing adolescent brain, specifically the parts responsible for impulsivity, attention, mood, and learning [6-8]. A few published case reports demonstrate that e-cigarettes can provoke seizures in previous non-epilepsy patients and increase seizure frequency in patients who have a history of seizures [9]. Furthermore, the FDA has been investigating the relationship between e-cigarette usage and seizures since April of 2019, as 114 cases of seizures in e-cigarette users were reported to the FDA between December 2010 and January 2019 [10]. As of March 1, 2021, more than 250 reports of e-cigarette associated seizures have been submitted, with approximately two third of cases occurring in youth or young adults [11]. As the reports provided limited data on the medical evaluation and clinical decision making involved in each case, the examination of these spontaneous reports to the FDA raised questions about causal links [11]. Therefore, detailed case reports with corroborating documentation are essential to clarify these questions.

## Case presentation

A 20-year-old man with a past medical history of allergic rhinitis presented to the clinic for his recurrent seizure after vaping nicotine evaluation. He has had three seizures after vaping nicotine in the past five years. He has also never used nicotine gum or patches. His first seizure was at the age of 15 years, which was a witnessed generalized tonic-clonic seizure occurring within minutes after he used his e-cigarette. He stopped vaping after his first seizure. Four years later while attending college, he had a second seizure that occurred shortly after taking several puffs of his vape pen in the bathroom before class had started. During this seizure, he was witnessed staring and suddenly falling to the ground, then whole body convulsing for approximately three minutes. He bit the lateral side of his tongue during the second seizure. He was hospitalized later that day with unremarkable CT head and labs, including a negative toxicology screen. He experienced postictal amnesia and fatigue for two days. Follow-up brain MRI and EEG one month later were unremarkable. He quit vaping after the second seizure.

However, three months later, the patient returned to vaping and had his third seizure within minutes of vaping. The seizure occurred while he was walking; he fell forward to the floor and had a generalized tonic-clonic seizure for 30 seconds. He experienced urinary incontinence during this seizure, and he was very confused and tired for at least 30 minutes afterward. His fall caused a head strike, which resulted in bruises on his forehead and around his eyes. A head CT without contrast, comprehensive drug screen, and labs were unremarkable. Considering cardiac etiology for the sudden onset loss of consciousness, cardiology was consulted, and his EKG and transthoracic echocardiogram (TTE) were normal. He was started on levetiracetam 500 mg PO BID (orally, twice a day). After the third seizure, the patient quit vaping and was started on a nicotine patch. He had not had any further seizures at his three-month clinic follow-up visit.

## Discussion

Seizure is caused by abnormal excessive or synchronous neuronal activity in the brain, leading to loss of consciousness, fall, generalized tonic-clonic convulsion, hypoventilation, hypoxemia, coma, and sudden death. Repeated and prolonged seizures can cause brain damage. The association between e-cigarette products and seizures is gradually unfolding in literature, as vaping has become a prominent public health issue in children and adolescents. In the past, nicotine toxicity occurred in adolescents who inadvertently chewed nicotine gum or were exposed to nicotine patches, but the source of nicotine toxicity has now evolved to e-cigarette products in an increasing number of young people [12]. According to U.S. Monitoring the Future surveys, nicotine vaping among middle and high school students has increased every year since 2016, and by 2019, 35% of high school seniors reported using e-cigarettes in the last year, with approximately one in four reported using in the last months and one in nine reported daily use [13]. In 2021, more than two million U.S middle and high school students reported current use of e-cigarettes, the most commonly used tobacco product by American youth [12]. E-cigarettes can contain as much nicotine as an entire pack of cigarettes, which explains why many e-cigarette consumers become addicted faster than regular cigarette consumers [14]. In a study about seizures and e-cigarette usage, researchers reported that 62% of reported seizures occur within 30 minutes of vaping, which suggests that seizures are caused by nicotine toxicity, as the level of inhaled nicotine is at its highest within this timeframe [15]. This theory is substantiated in this patient, as his three seizures occurred within minutes of vaping.

Nicotine and its receptors play an important role in cerebral neural activity, as autosomal dominant nocturnal frontal lobe epilepsy (ADNFLE) is associated with the mutation of nicotinic acetylcholine receptors. At the toxicity level, nicotine may alter neuronal excitability and lower the seizure threshold. Nicotinic acetylcholine receptors are highly expressed in the neocortex, amygdala, hippocampus, and thalamus, where they modulate neuronal excitability [16,17]. Exogenous nicotine activates nicotinic acetylcholine receptors (nAChRs) in the neocortex, amygdala, hippocampus, and thalamus, which could generate seizures by facilitating and synchronizing spontaneous oscillations in thalamo-cortical circuits by increasing glutamate release [16,17].

Considering the facts that 1) this patient had no past medical history or family history of seizures and no other seizure risk factors, 2) all three seizures occurred within minutes after vaping when the nicotine level is at its highest, we believe that the seizures in this patient were provoked by vaping.

## Conclusions

In conclusion, we report a case of recurrent seizure provoked by e-cigarette use. This patient had no past medical history or family history of seizures and no other seizure risk factors. Additionally, all three seizures occurred within minutes after vaping when the nicotine level is at its highest, leading to the conclusion that his three seizures were provoked by vaping. Given the young age of this patient and his onset of vaping during high school, it is vital that healthcare providers educate youth, young adults, and their caregivers on the health effects of vaping, especially seizures due to their life-threatening consequences.
